# Using a Call Center to Reduce Harm From Self-Administration of Reproductive Health Medicines in Bangladesh: Interrupted Time-Series

**DOI:** 10.2196/12233

**Published:** 2019-08-04

**Authors:** Katherine Keenan, Katharine Footman, Munnaf Sadekin, Kate Reiss, Reena Yasmin, Hannah Franklin, Kathryn Church

**Affiliations:** 1 University of St. Andrews School of Geography and Sustainable Development St. Andrews United Kingdom; 2 Marie Stopes International London United Kingdom; 3 Marie Stopes Bangladesh Dhaka Bangladesh; 4 London School of Hygiene and Tropical Medicine London United Kingdom

**Keywords:** reproductive health, call center, Bangladesh, menstrual regulation, abortion, induced, abortion, legal, health behavior, help-seeking behavior

## Abstract

**Background:**

Annually, there are approximately 25 million unsafe abortions, and this remains a leading cause of maternal morbidity and mortality. In settings where abortion is restricted, women are increasingly able to self-manage abortions by purchasing abortion medications such as misoprostol and mifepristone (RU-486) from pharmacies or other drug sellers. Better availability of these drugs has been shown to be associated with reductions in complications from unsafe abortions. In Bangladesh, abortion is restricted; however, menstrual regulation (MR) was introduced in the 1970s as an interim method of preventing pregnancy. Pharmacy provision of medications for MR is widespread, but customers purchasing these drugs from pharmacies often do not have access to quality information on dosage and potential complications.

**Objective:**

This study aimed to describe a call center intervention in Bangladesh, and assess call center use over time and how this changed when a new MR product (combined mifepristone-misoprostol) was introduced into the market.

**Methods:**

In 2010, Marie Stopes Bangladesh established a care provider–assisted call center to reduce potential harm from self-administration of MR medications. The call center number was advertised widely in pharmacies and on MR product packaging. We conducted a secondary analysis of routine data collected by call center workers between July 2012 and August 2016. We investigated the reported types of callers, the reason for call, and reported usage of MR products before and after November 2014. We used an interrupted time series (ITS) analysis to formally assess levels of change in caller characteristics and reasons for calling.

**Results:**

Over the 4-year period, 287,095 calls about MR were received and the number of users steadily increased over time. The most common callers (of 287,042 callers) were MR users (67,438, 23.49%), their husbands (65,999, 22.99%), pharmacy workers (65,828, 22.93%), and village doctors (56,036, 19.52%). Most MR calls were about misoprostol, but after November 2014, a growing proportion of calls were about the mifepristone-misoprostol regimen. The most common reasons (of 287,042 reasons) for calling were to obtain information about the regimen (208,605, 72.66%), to obtain information about side effects (208,267, 72.54%), or to report side effects (49,930, 17.39%). The ITS analyses showed that after November 2014, an increasing number of calls were from MR users who had taken the complete regimen (*P*=.02 and who were calling to discuss reported side effects (*P*=.01) and pain medication (*P*=.01), and there were fewer calls asking about dosages (*P*<.001).

**Conclusions:**

The high call volume suggests that this call center intervention addressed an unmet demand for information about MR medications from both MR users and health care providers. Call center interventions may improve the quality of information available by providing information directly to MR users and drug sellers, and thus reducing the potential harm from self-management of MR medications.

## Introduction

### Background

The most recent estimates suggest that between 2010 and 2014, there were 56 million abortions annually, of which 25 million were unsafe [1]. Unsafe abortions are a leading cause of maternal morbidity and mortality and are particularly prevalent in low-income settings. In settings where abortion is restricted or difficult to access, women are increasingly able to self-manage abortions more safely by purchasing abortion medications such as misoprostol and mifepristone from pharmacies or other drug sellers [2,3]. The increasing availability of these medications is thought to have resulted in reduced morbidity from unsafe self-induced abortion in many low-income countries [4-6]. The World Health Organization’s (WHO) guidance on health worker roles for safe abortion states that women can safely self-manage elements of medical abortion in circumstances where women have a source of accurate information and access to a health care provider should they need or want it [7]. However, women accessing these medications from pharmacies often do not receive adequate information from pharmacy workers because pharmacy staff lack knowledge about effective regimens and potential complications [2,3].

The increasing use of mobile and wireless technologies for health—known as mobile health (mHealth)—has huge potential to improve health systems in low- and middle-income countries (LMICs) through better access to knowledge and information [8]. mHealth approaches are being promoted in Bangladesh, where household mobile phone ownership has risen rapidly from 32% of households in 2007 to 89% in 2014 [9,10]. Call centers and hot lines can help to reduce geographic inequalities in health services and may be particularly appropriate for reproductive health issues because they can provide client anonymity when discussing sensitive matters. Recent studies have demonstrated the importance of hotlines for enabling women’s access to information on safe abortion medications in legally restrictive settings such as Latin America and Indonesia [11,12], and safe abortion hotlines exist in more than 20 countries in the global South [13]. Furthermore, it has been shown that mHealth approaches (involving getting advice and support by phone or the internet) can be successfully used by women self-administering medical abortions [14]. However, it is recognized that more research is needed to understand exactly how women use abortion hotlines for support and for what reasons [15].

### The Context of Menstrual Regulation in Bangladesh

In Bangladesh, abortion is legal only to save a woman’s life; however, menstrual regulation (MR), “an interim method of establishing non-pregnancy in women at risk of being pregnant,” was introduced in the country in 1972 as a strategy to reduce morbidity and mortality from unsafe abortion [16]. Pregnancy is not confirmed before administering the procedure or medications. The approved methods for MR services are manual vacuum aspiration (MVA) up to 12 weeks after a missed period and, since 2013, administration of mifepristone and misoprostol up to 9 weeks [17]. In February 2013, the first combination pack of mifepristone-misoprostol (branded the MM Kit) was accepted by the National Drug Administration of Bangladesh and came onto the market as an alternative to surgical MR. A second brand, the MTP Kit, was approved and became available later in the year [18]. Self-induced MR with medication has also long been practiced in Bangladesh through off-label use of misoprostol, which is registered for use for postpartum hemorrhage and peptic ulcer, provided through pharmacies [19]. There is little regulation of pharmacies in Bangladesh, and many medications are sold over the counter without a prescription [20], including misoprostol and MR medications [19,21]. Since the registration of the combination pack of mifepristone-misoprostol, combination products as well as misoprostol alone have increasingly become available in pharmacies [21]. Information provided by the Directorate General of Drug Administration in Bangladesh shows that currently 32 brands of misoprostol only are registered for sale by 28 pharmaceutical companies, and the combination regimen is registered for sale by at least 8 different companies [22]. However, as seen in other countries, pharmacy and mystery client surveys conducted in Bangladesh have documented poor knowledge of effective regimens for medical MR among pharmacy workers [19,21,23]. This is particularly important, given that misoprostol is not registered for MR and does not contain instructions for use for this indication.

The most recent study of MR incidence in Bangladesh showed that in 2014, an estimated 430,000 MR procedures (using MVA or medication) were performed in health facilities nationwide, whereas an estimated 1,194,000 induced abortions occurred and 257,000 women were treated for complications from abortion [24]. Singh et al found that of the women presenting with postabortion complications, the proportion of women with hemorrhage increased substantially between 2010 and 2014, from 27% to 48%, and the estimated proportion with incomplete abortion declined from 66% to 56% [24]. This is consistent with a pattern of increased use of misoprostol-induced abortion, in which heavy and prolonged bleeding is a common side effect. It is possible that this symptom would have resolved without further treatment for some women, but the trend suggests the need for improved access to information about correct use and potential side effects and complication management.

### The Marie Stopes Bangladesh Call Center Initiative and Aims of the Study

Marie Stopes Bangladesh (MSB) is a sexual and reproductive health service provider, operating through 600 service delivery outlets across all 64 districts of Bangladesh, including static centers, outreach teams accessing hard-to-reach and underserved rural communities, public sector support, and social marketing channels. To address the lack of access to accurate information about MR medications, MSB set up a call center in 2010 with the goal of preventing potential harm for individuals who purchase MR medications from pharmacies and drug shops. This study uses routine data to investigate the changing use of the call center over a 4-year period (2012-2016). The objectives of the study were to (1) describe the characteristics of call center users and their reasons for calling and (2) assess whether the introduction of a mifepristone-misoprostol combined regimen into the market led to a change in the usage of the call center. It is hoped that the results can inform future programming for information provision about mifepristone and misoprostol in Bangladesh and other LMICs. The term pharmacy can refer to a range of businesses of various sizes and legal statuses, and in this paper, we refer to pharmacies and drug shops as any outlet whose business is selling medicines, regardless of their training, staff qualifications, or legal status. We refer to mifepristone-misoprostol and misoprostol alone as *MR medications* for the remainder of the paper.

## Methods

### Details of the Call Center Initiative

**Figure 1:**
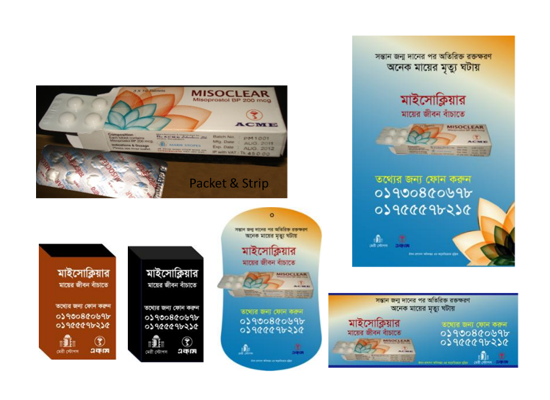
Examples of call centre promotional materials.

This call center initiative is one of 27 reproductive health call centers supported by Marie Stopes International worldwide [25]. The call center provides a supplementary information service for MR users and is open 24 hours a day, 7 days a week. The call charge is at the rate of a regular call to a mobile phone, but callers can also request an immediate free callback. The call operators are all female, mid-level medical service providers who have completed a 36-month course from the Medical Assistant Training School. All operators receive in-house training covering counseling skills for MR and related reproductive health topics, MSB services, the use of medication for MR, management of side effects and complications, referring to the nearest private or public facility, and the legal status of MR. The operators are also trained to discuss contraceptive options with callers who have used or are intending to use medications for MR. Regular refresher training sessions are conducted for call operators. The quality of calls is routinely monitored using mystery callers. The call center number is widely promoted through a variety of channels; MSB distributes promotional stickers, wallet-sized cards, and posters through pharmacies and village doctors and promotes the call center verbally when attending pharmacies and during orientations of drug sellers and village doctors (eg, see Figure 1). In addition, the call center number was printed on the packaging and foil of 2 MSB products (one misoprostol only from 2012 and another combination pack [mifepristone-misoprostol] from 2014) and a non-MSB combination pack product from 2015.

### Data

This study retrospectively analyzed routine data collected by MSB call center operators between July 2012 and August 2016. In June 2012, a 15-item monitoring form was designed to collect information on the profile of callers and their reasons for calling. All operators were trained to administer the questions from the monitoring form during every call, to record responses on paper during the call or immediately after, and to enter the data into an Epi Info 7 database (developed by the Centers for Disease Control and Prevention) between calls. No identifying information such as the caller’s name or address was collected. Each caller was asked if they had called before but repeat callers are treated as distinct individuals and their records from previous calls are not matched. Data collection commenced in July 2012 and is ongoing.

### Variables and Analysis

The variables in the monitoring form are as follows. Callers were asked about their location (one of several divisions of Bangladesh), the age range of the MR user (in 5-year groups), and whether they were a repeat caller (yes or no). Operators recorded the caller’s relationship to the MR user with the following response options: pharmaceutical representative, pharmacist, pharmacy worker, village doctor, woman herself (MR user), husband, mother, and other relative or friend of MR user). All callers were asked the general reason for calling, and the call center operator could select multiple responses from a list of 12 (including misoprostol for medical MR, misoprostol for other conditions, combination regimen for medical MR, family planning, other reproductive or general health questions, or information on the nearest clinic or hospital). If the call was MR related, the specific queries of the caller were recorded (including whether misoprostol can be used for medical MR, correct dosage for medical MR [timing, dosage, or route], questions about side effects and complications, experience of side effects, experience of complications, taken the wrong dose, pain medications, and accessing the clinic or hospital). If the reason for calling was that the end user was experiencing side effects or suspected complications, the types of side effects and suspected complications were recorded. Complications are reported as *suspected* because they were diagnosed remotely by the call center operator based on information given by the caller and may not be clinically accurate. MR-related callers were asked whether the MR drug had already been purchased, the brand purchased, and whether the end user had already taken the medication.

Some adaptations were made to the monitoring form after the initiation of data being recorded: the repeat caller variable was only included from July 2013, whether the MR drug had already been purchased was only available from November 2014, brand data were available only from September 2013, and under general reasons for calling, the response options *mifepristone-misoprostol combination pack*, *general health*, and *other reproductive health* were only available from November 2014. The analysis received ethical approval from the Marie Stopes Independent Ethics Review Committee (012‐15) and the Bangladesh Medical Research Council (803). The ethics committee approved our analysis of routine data without explicit consent from the participants to use their data for research purposes, for the following reasons: (1) the risks of identification of callers is minimal, especially as all data are presented in aggregate, (2) the data were not part of a research project but part of routine monitoring, and (3) the medications used were part of a national protocol rather than being introduced as part of the study.

### Statistical Analysis

#### Use of the Call Center

We conducted descriptive analyses of the number of calls, profiles of call center users, and their reported reasons for calling over time, using aggregate monthly data.

#### Differences in Call Center Use

To assess whether the introduction of the combination regimen was associated with differences in call center use, we compared caller characteristics before and after November 2014. This month was chosen because it was when the call center number started to be printed on a combination pack, so although the combination regimen was available on the market from March 2013, the call center was unlikely to receive calls until the call center number was widely advertised through product packaging. Interrupted time series (ITS) analysis was used [26] to formally evaluate whether changes in the number of calls according to reason for calling and caller type had occurred after November 2014. We necessarily restricted the ITS to outcomes that had complete data across the entire period. As MR combination regimen users receive more comprehensive dosage instructions from the packaging than MR misoprostol-only users, we hypothesized that the introduction of the combination regimen would result in a change in both the level and slope of the relationship between types of callers, reason for calling, and whether the MR user had taken the drug before calling in the pre- and postintervention periods. We conducted the ITS analysis using STATA 14.0 [27] using the STATA module ITSA: Stata module to perform ITS analysis for single and multiple groups [28].

### Misoprostol-Only and Combination Regimen Callers

We also compared characteristics of calls related to misoprostol only and the combination pack using bivariate crosstabulations.

## Results

### Use of the Call Center

**Figure 2:**
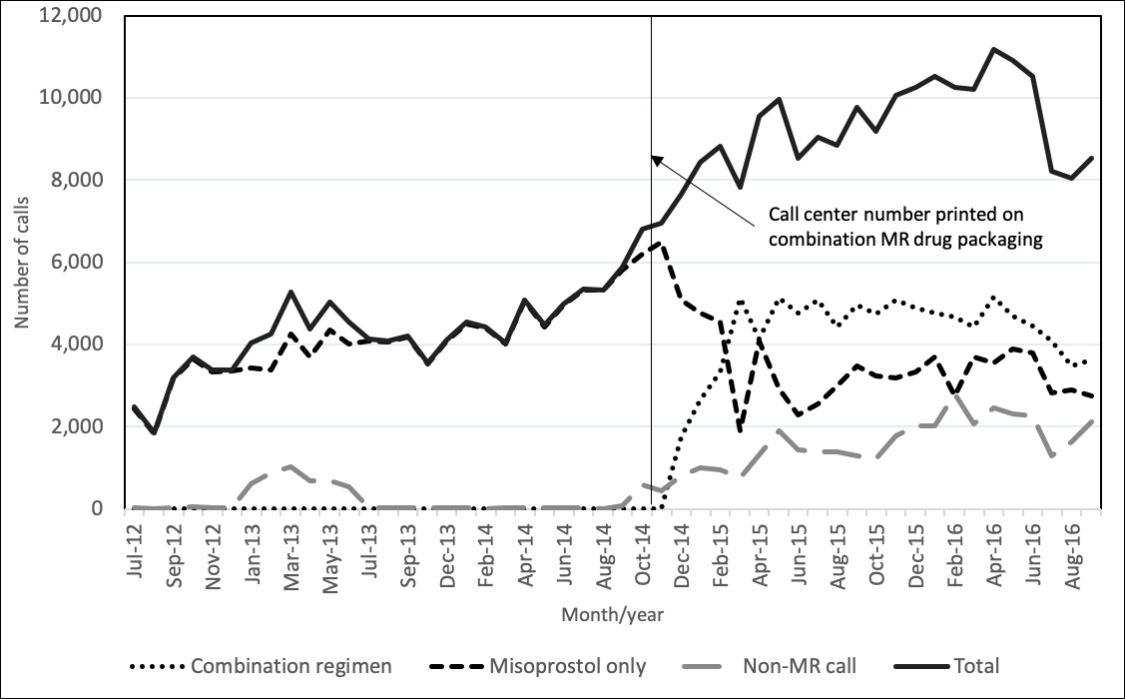
Number of calls received by the Marie Stopes Bangladesh call center, July 2012 to August 2016. MR: menstrual regulation.

A total of 344,827 calls were made to the call center between July 2012 and August 2016, and the number of calls per month increased from 2778 in July 2012 to a peak of 11,157 calls in March 2016, falling to 8516 in August 2016 (Figure 2). Over the whole period studied, 83.25% of the calls were about MR (either misoprostol or combination regimen; n=287,095). Between July 2012 and October 2014, all MR calls were about misoprostol, but after the introduction of the call center number to a combination pack in November 2014, calls about the combination regimen grew rapidly and quickly overtook the number of calls about misoprostol only MR.

The number and proportion of calls that were for non-MR queries also increased after November 2014, and by August 2016, they accounted for a quarter of all calls. Non–MR-related reasons for calling included questions about accessing the nearest clinic or hospital (28,554/344,827, 8.28%), family planning (6403/344,827, 3.10%), other reproductive or general health topics (7156/344,827, 3.47%), misoprostol for other indications (1518/344,827, 0.49%), and other reasons (21,114/344,827, 10.06%). Non–MR-related calls are excluded from the subsequent analysis.

### Characteristics of Those Calling About Menstrual Regulation

The characteristics of MR-related calls are shown in Table 1. Over half (163,018, 56.79%) of the 287,042 MR-related calls were made by MR users, their friends, or relatives: 67,438 (23.49%) from the MR user, 65,999 (22.99%) from their husbands, 17,115 (5.99%) from their mother or other relatives, and 12,446 (4.34%) from friends. Health care or medication providers represented 123,467 (43.01%) of calls, 65,828 (22.93%) were from pharmacists or pharmacy workers, 56,036 (19.52%) of calls were from village doctors, and a small number of calls (1603, 0.56%) were received from pharmaceutical representatives. Most calls (141,148, 49.17%) came from the two most populous divisions of Bangladesh, Dhaka and Chittagong. The majority of callers reported the MR user’s age as being below 25 years.

Just under half of MR calls (110,635/250,814, 44.94%) were from repeat callers. We cross-tabulated repeat callers with other characteristics (results available on request) and found that repeat callers were more likely to be MR users or pharmacy workers compared with calls from first-time callers, but there were no other significant differences in the characteristics of repeat callers and first-time callers. We also cross-tabulated the reason for calling with the type of caller, which showed that that calls about misoprostol for indications other than MR (eg, related to postpartum hemorrhage) were more likely to be made by a provider (predominantly driven by calls from village doctors) than an MR user or their family.

Of 287,095 calls, 109,429 (64.50%) calls were made when the MR user had already purchased the drug, and 68,732 (23.95%) of the MR users had taken MR medications before calling. The most common MR-related reasons for calling were to ask if medications could be used for MR (161,145/169,453, 96.75%), to obtain regimen information (208,605/287,095, 72.66%), and to get information about side effects (208,267,287,095, 72.54%). Overall, 49,930 of 287,095 people (17.39%) called because they were experiencing side effects, 21,207 (7.39%, 21,207/218,738) called to ask about pain medication, 6745 (3.08%, 6745/218,738) suspected they had taken the wrong dose, and 11,207 (3.90%, 11,207/287,095) wanted information on accessing the nearest clinic or hospital. The most common side effects reported were cramps (7.26%) followed by bleeding (4.06%) and diarrhea (4.10%), fever (2.84%), and vomiting (1.14%; data not shown). In addition, 2.83% of MR callers said they thought they were experiencing complications, and almost all of these were suspected to have incomplete MR. Only 3 callers reported excessive blood loss. Most callers called for more than one reason.

**Table 1:** Characteristics of individuals calling the Marie Stopes Bangladesh call center about menstrual regulation (MR) from July 2012 to August 2016.

Characteristic of the call or caller		July 2012-October 2014, %	November 2014-August 2016, %	Total, n (%)	*P* value
**Type of caller**					<.001
	Pharmaceutical representative	0.51	0.59	1603 (0.56)	
	Pharmacist or pharmacy worker	21.83	23.70	65,828 (22.93)	
	Village doctor	20.76	18.67	56,036 (19.52)	
	MR user (woman)	21.70	24.74	67,438 (23.49)	
	Husband of MR user	28.19	19.39	65,999 (22.99)	
	Mother or other relative of MR user	4.08	7.27	17,115 (5.96)	
	Friend of MR user	2.61	5.54	12,466 (4.34)	
	Don’t know, refuse and missing	0.33	0.10	557 (0.19)	
**Reported age of MR user (years)**					<.001
	<20	20.20	25.85	67,579 (23.54)	
	20-24	40.04	35.03	106,453 (37.08)	
	25-29	25.67	21.18	66,074 (23.01)	
	30 or older	9.24	14.35	35,188 (12.26)	
	Don’t know, refuse and missing	4.85	3.60	11,801 (4.11)	
**Division of Bangladesh**					<.001
	Dhaka	28.47	27.27	79,700 (27.76)	
	Rajshahi	10.72	12.54	33,870 (11.80)	
	Rangpur	9.01	10.37	28,175 (9.81)	
	Chittagong	22.21	20.84	61,448 (21.40)	
	Sylhet	8.80	11.25	29,422 (10.25)	
	Khulna	8.44	7.73	23,017 (8.02)	
	Barisal	6.58	7.09	19,758 (6.88)	
	Out of country, don’t know, refuse and missing	5.76	2.91	11,705 (4.08)	
**Repeat caller^a^**					<.001
	Don’t know and refuse	12.44	8.21	23,450 (9.53)	
	Yes	45.69	44.61	110,635 (44.94)	
	No	41.87	47.18	112,085 (45.53)	
**MR user had purchased drug before calling^b^**					—^c^
	Don’t know and refuse	—	—	1070 (0.63)	
	Yes	—	—	109,429 (64.50)	
	No	—	—	59,158 (34.87)	
**MR user had taken drug before calling**					<.001
	Taken complete regimen	12.38	17.80	44,745 (15.59)	
	Started the regimen	10.17	7.10	23,987 (8.36)	
	Not taken	72.78	72.35	208,211 (72.52)	
	Don’t know and other	4.67	2.75	10,152 (3.54)	
**Specific MR-related query^d^**					
	Whether misoprostol used for MR^e^	—	96.75	164,145 (96.75)	—
	Correct MR dosage and regimen^f^	73.11	72.35	208,605 (72.66)	<.001
	Information on side effects and complications	72.82	72.35	208,267 (72.54)	<.001
	Experiencing side effects	15.12	18.96	49,930 (17.39)	<.001
	Caller suspects complications	2.04	3.37	8118 (2.83)	<.001
	Caller thinks they have taken the wrong dose^g^	4.22	2.75	6745 (3.08)	<.001
	Questions about taking pain medication	6.75	7.83	21,207 (7.39)	<.001
	Questions about accessing clinic or hospital	1.04	5.88	11,207 (3.90)	<.001
Total		40.91	59.09	287,095 (100.00)	—

^a^Question introduced in July 2013.

^b^Missing data due to question being introduced in November 2014.

^c^Not applicable.

^d^Multiple responses allowed, so column percentages can total more than 100.

^e^Missing data due to question being introduced in November 2014.

^f^Combined responses to queries about timing, dosage, and route.

^g^Question not asked in September 2013-October 2014.

Table 1 also compares the characteristics of callers before and after November 2014 when the call center number started to be printed on the combination pack. As expected, because of the large population size, all differences were statistically significant using the chi-square test. After November 2014, a larger proportion of calls were from pharmacy workers, MR users, and their relatives, whereas proportionally fewer calls were from MR users’ husbands and village doctors. After November 2014, more callers reported taking the complete regimen before calling and proportionally more reported experiencing side effects, but these trends are likely linked.

### Change in Call Center Use Over Time

**Figure 3:**
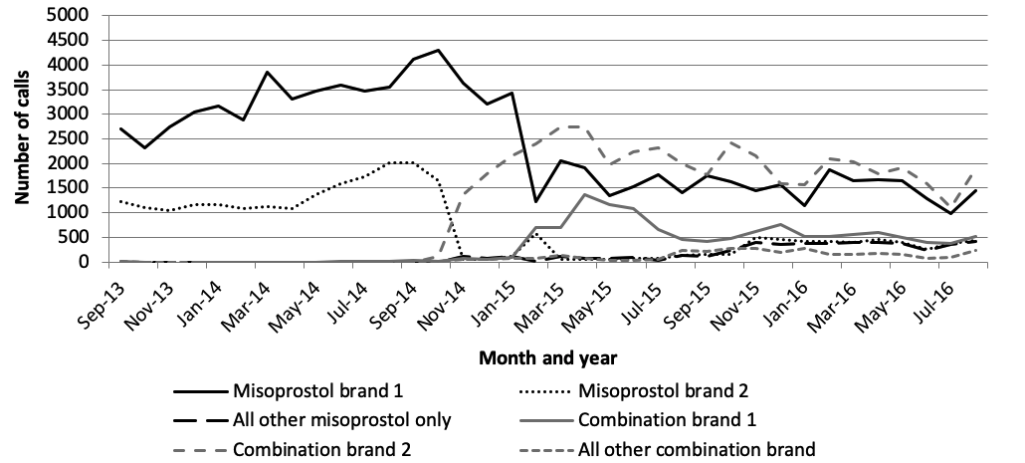
Number of calls by month according to brand of menstrual regulation drug purchased (September 2013-August 2016).

The brands of MR medications reported to have been purchased by call center users varied over time, and details of the most commonly mentioned brands are shown in Table 2. Figure 3 shows the number of calls by type and brand of MR drug over time, grouped by the brands in Table 2. Before November 2014, the callers were mainly using 2 brands of misoprostol. When the call center number was advertised on the packaging of a combination pack released into the market in November 2014, an increasing number of calls were related to this brand of the combination regimen (brand 2). In January 2015, the combination regimen brand 1 also began to advertise the call center number on its packaging (although the brand had been on the market since February 2013), and there was a corresponding increase in calls. The call center also received calls about products that do not have the call center number on the packaging, reflecting the promotion of the call center through other channels including in-pharmacy posters and danglers.

Time series plots showed that over the 4-year reference period, the mean number of MR users who reported they were under 20 years increased. Over time, there was a steady decrease in calls from husbands of MR users, and slight increases in calls from MR users and pharmacists or pharmacy workers. There was a noticeable increase in the proportion of callers who reported having taken the complete regimen and decrease in those who had called the call center when they had only started the regimen.

**Figure 4:**
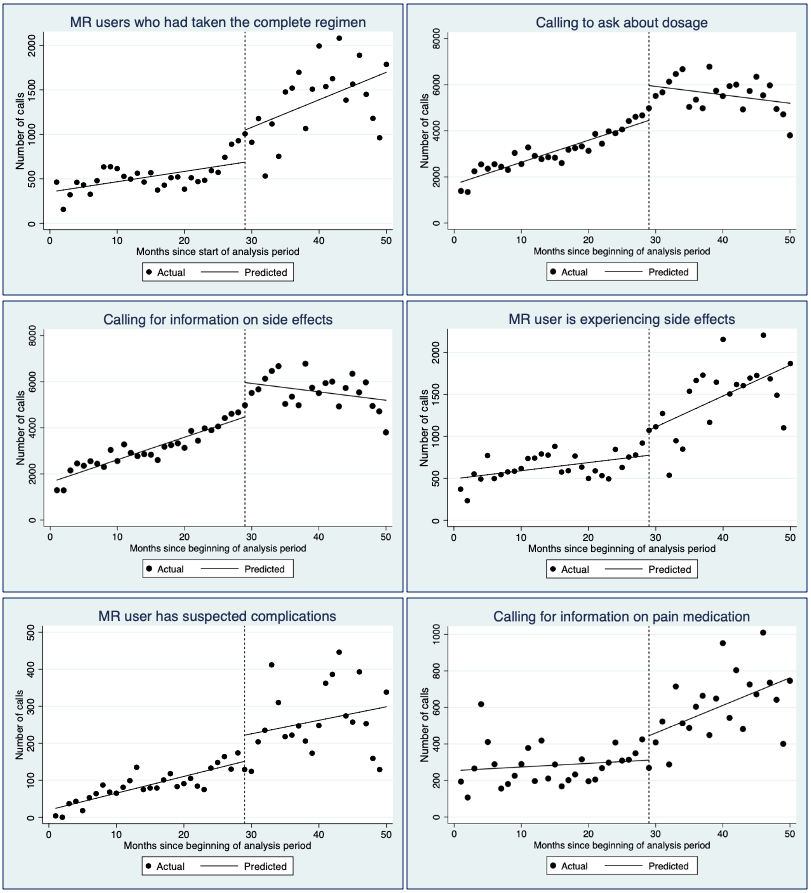
Plotted time series data and fitted regression lines considering pre- and postintroduction of the combination regimen (November 14) for selected outcomes.

ITS analyses using November 2014 as the intervention point are shown in Table 3 as regression results and Figure 4 as plots for selected outcomes (plots for all outcomes in Table 3 available on request). Table 3 shows that there were significant differences in the trends in the number of calls at November 2014 (the last column) related to several factors. After November 2014, an upward trend began in the number of calls where the user had taken the complete regimen, and likely related to this, there were similar post-November 2014 upward trends in calls concerning MR users experiencing side effects and enquiring about pain medication. There were downward trends post-November 2014 in the number of calls asking about dosage and regimen and asking for information on side effects. There were no significant trend changes in the caller age and types of callers and in the number of calls regarding suspected complications.

**Table 2:** Details of the most commonly mentioned menstrual regulation medications.

Product	Date registered in Bangladesh	Date call center number first printed on product
Misoprostol brand 1	September 2011	September 2011
Misoprostol brand 2	2002	Not printed
Combination brand 1	February 2013	January 2015
Combination brand 2	October 2014	October 2014

**Table 3:** Interrupted time series regression results comparing the number of calls pre- and post-November 2014, overall, by type of caller, and reason for calling.

Characteristic of the call or caller		Mean calls per month at start of time series	Time trend (calls per month) before November 2014 (95% CI)	Step change at November 2014 (95% CI)	Change in time trend after November 2014 (95% CI)
Overall		2641.5	115.0 (87.3 to 142.8)	1866.6 (1064.5 to 2668.8)	−1.6 (−0.73 to 70.0)
**Type of caller**					
	Pharmacist or pharmacy worker	352.2	41.7 (32.2 to 51.2)	277.5 (10.6 to 544.5)	2.8 (−15.5 to 21.2)
	Menstrual regulation (MR) user (woman)	732.4	13.1 (−5.5 to 31.8)	632.7 (60.8 to 1204.8)	16.5 (−21.3 to 54.5)
**Reported age of MR user (years)**					
	<20	362.7	35.8 (28.1 to 43.7)	391.8 (75.0 to 708.6)	22.3 (−2.8 to 47.4)
**MR user had taken drug before call**					
	Taken complete regimen	362.7	11.6 (3.1 to 20.1)	360.8 (46.8 to 674.8)	30.9 (4.4 to 57.4)
**Reason for calling about MR**					
	Correct MR dosage and regimen	1775.7	95.6 (77.2 to 114.0)	1510.5 (771.1 to 2250.0)	−36.9 (−96.8 to 23.7)
	Information on side effects and complications	1733.8	97.8 (79.2 to 116.5)	1490.5 (754.2 to 2226.9)	−36.5 (−96.8 to 23.7)
	Experiencing side effects	503.5	9.7 (2.4 to 16.9)	300.3 (−24.2 to 624.9)	36.9 (10.7 to 63.2)
	Caller suspects complications	24.2	4.5 (3.4 to 5.7)	70.6 (−23.3 to 164.6)	3.7 (−4.3 to 11.6)
	Questions about pain medication	256.2	2.0 (−4.8 to 8.8)	133.1 (−11.0 to 278.2)	15.1 (3.3 to 26.9)

### Misoprostol-Only and Combination Regimen Callers

Table 4 compares the characteristics and reasons for calling between callers who had purchased misoprostol-only versus the combination pack (data from November 2014 to August 2016). There was little difference in the age profile of the 2 types of callers. Combination pack callers were more likely to be MR users themselves (26.9% vs 22.0%) and less likely to be the husbands of MR users (18.0% vs 21.2%). Combination pack purchasers were more likely than misoprostol-only purchasers to call after taking the complete regimen (19.0% vs 16.3%), to have called for information about dose or timing (73.6% vs 70.8%), or to say they were experiencing side effects (19.6% vs 18.1%). Misoprostol-only callers were more likely to need information about pain medication (8.7% vs 7.2%), think they had taken the wrong dose (3.6% vs 2.1%), or need to access the clinic or hospital (7.1% vs 5.0%). Combination pack callers were more likely to call citing bleeding as a side effect (6.3% vs 2.8%), which may be because more had taken the complete regimen or may reflect underdosing of misoprostol only users.

**Table 4:** Differences between combination drug and misoprostol-only purchasers (November 2014-August 2016, N=169,619).

Characteristic of call or caller		Combination regimen (n=95,447), n (%)	Misoprostol only (n=74,210), n (%)	*P* value
**Type of caller**				.001
	Pharmaceutical representative	523 (0.55)	481 (0.65)	
	Pharmacist or Pharmacy worker	21,993 (23.05)	18,207 (24.54)	
	Village doctor	17,920 (18.78)	13,740 (18.52)	
	Menstrual regulation (MR) user (woman)	25,648 (26.88)	16,315 (21.99)	
	Husband of MR user	17,156 (17.98)	15,741 (21.22)	
	Mother and other relative of MR user	6919 (7.25)	5411 (7.29)	
	Friend of MR user	5163 (5.41)	4236 (5.71)	
	Don’t know, refuse and missing	103 (0.11)	63 (0.08)	
**Reported age of MR user (years)**				.001
	<20	25,625 (26.85)	18,232 (24.57)	
	20-24	32,454 (34.00)	26,973 (36.35)	
	25-29	20,127 (21.09)	15,799 (21.29)	
	30 or older	13,748 (14.40)	10,593 (14.27)	
	Don’t know, refuse and missing	3493 (3.66)	2613 (3.52)	
**Repeat caller^a^**		42,855 (44.90)	32,821 (44.23)	.001
**MR user had taken drug before calling**				.001
	Taken complete regimen	18,099 (18.96)	12,106 (16.31)	
	Started the regimen	5097 (5.34)	6947 (9.36)	
	Not taken	70,224 (73.57)	52,519 (70.77)	
	Don’t know and other	2027 (2.12)	2638 (3.55)	
**Specific MR-related query^b^**				
	Whether misoprostol or combination pack used for MR	93,468 (97.93)	70,677 (95.24)	.001
	Correct MR dosage and regimen	70,224 (73.57)	52,523 (70.78)	.001
	Information on side effects or complications	70,224 (73.57)	52,523 (70.78)	.001
	Experiencing side effects	18,750(19.64)	13,422 (18.09)	.001
	Caller suspects complications	2942 (3.08)	2783 (3.75)	.001
	Thinks they have taken the wrong dose^c^	2024 (2.12)	2650 (3.57)	.001
	Questions about pain medication	6854 (7.18)	6431 (8.67)	.001
	Accessing clinic or hospital	4739 (4.97)	5245 (7.07)	.001
**Type of side effect reported^d^**				
	Nausea	757 (0.79)	508 (0.68)	.01
	Vomiting	1489 (1.56)	1308 (1.76)	.001
	Diarrhea	4136 (4.33)	3376 (4.55)	.03
	Headache	1937 (2.03)	589 (0.79)	.001
	Cramps	6850 (7.18)	6379 (8.60)	.001
	Bleeding	5981 (6.27)	2041 (2.75)	.001
	Fever	2009 (2.10)	2377 (3.20)	.001

^a^Question introduced in July 2013.

^b^Multiple responses are allowed, so percentage may sum to more than 100%.

^c^No data available in September 2013-October 2014.

^d^Question introduced in July 2013.

## Discussion

### Principal Findings

This study analyzed the usage patterns of a call center in Bangladesh that was established to reduce harm from potential incorrect use of MR medications, and the findings point to the effectiveness and feasibility of this approach. Consistent with findings from surveys of pharmacy workers undertaken in 2011 [19] and in 2013 [21], the results suggest that misoprostol and, more recently, the combination regimen are being used widely for MR but that gaps in knowledge exist among both end users and drug providers. There was a high and increasing number of calls about MR over the 4-year period, suggesting that the call center addressed an unmet need for information. MR users and their relatives, and providers or sellers of medications most often called for information about the correct dosage, and the potential side effects or complications of MR medications. Changes in the types of drugs available for MR were associated with changes in call center use. The high call volume and high proportion of repeat calls suggests that the call center method of communicating information on this stigmatized issue is acceptable to both providers and end users and their relatives, including younger women, and that mHealth initiatives can successfully reach women, providers, and their relatives of different ages and geographical areas of Bangladesh [29].

This study adds to the evidence on call center support for maternal and reproductive health issues [12,29,30] by detailing the experience in Bangladesh, where availability of information on MR medications remains insufficient.

Improving the quality of information available to women who purchase mifepristone or misoprostol from pharmacies is a challenge, and although pharmacy worker–focused interventions such as training and detailing have had some impact on pharmacy provision [3,31,32], these interventions can be expensive and difficult to provide at scale. In addition, they may not be appropriate in contexts where staff turnover at pharmacies is high [2]. Our analysis suggests that in addition to making information available to the end user directly, a call center intervention can make information available to those selling MR medications or advising local communities on health issues. The potential to reduce harm from incorrect use of MR medications is reflected in the fact that the majority of callers made contact before taking the products. That nearly one-fifth of calls were from village doctors deserves further investigation on the role of these health providers on providing MR and reproductive health care. Although a lot of current research concentrates on pharmacies as frontline providers, village doctors may be another way of improving access to quality care in hard-to-reach populations.

An important function of the call center is to provide referral information to women experiencing complications after taking misoprostol for MR. The WHO recommended a misoprostol-only MR regimen of 800 mcg taken sublingually up to 3 times at 6-, 12-, and 24-hour intervals [33] has been found to have an 85% to 90% effectiveness rate [34,35]. If callers to the MSB call center were representative of misoprostol-only MR users, we would therefore expect a minimum of 15% of women to have experienced an incomplete MR. Of misoprostol-only callers to the call center, 2.7% reported that the end user was experiencing suspected complications, all of which were suspected incomplete procedures. This suggests that most women who are experiencing complications or incomplete MR are not calling the call center for support. It is possible that these women may be going directly to clinics or back to the drug seller to receive follow-up care, but further research into the care-seeking patterns of women who experience incomplete MR after using misoprostol alone is required.

Unsurprisingly, the introduction of the combination pack into the market was followed by a decline in the number of calls regarding misoprostol-only MR and an increase in calls about the combination regimen. As increases in calls were clearly linked to when various brands started to include the call center number on the packaging, it also suggests the efficacy of this technique to publicize call centers as a source of support for women self-administering MR medications. Unlike misoprostol, the combination regimen is packaged for MR with instructions on use. ITS analysis showed that after the call center number started to be printed on combination regimen packaging, significantly more calls were made about MR users who had taken the complete regimen and who were experiencing side effects and wanted information on pain medication. Conversely, downward trends were seen after November 2014 for enquires about dosage, regimen, and asking for information on side effects, which is logical given that the combination regimen pack prints usage instructions. This suggests that after the introduction of the combination regimen to the market, callers more often called later in the MR process and with different kinds of queries.

Our sample is likely not representative of all women who are accessing MR medications through pharmacies in Bangladesh, and preliminary analyses suggest that call center users may be younger than the general population of MR users in Bangladesh. As a crude comparison of age distribution, we used the 2014 Bangladesh Demographic and Health survey (DHS) [9] to calculate the age distribution of women who reported having used MR is the previous 3 years (N=538) and compared this with the reported age distribution of MR users reported to call center operators. The age profile of recent MR users from the DHS is notably older, with the majority (87.7%) being 25 years and older; and the majority of users reporting being between 30 and 34 years. Although the differing age distributions could be partly related to the DHS reference period of 3 years or underreporting of MR by younger women in the DHS, it could also suggest that MR users contacting the call center may be on average younger than the general population of MR users. This may reflect younger women being more likely to access MR using medications, to access it through pharmacies, to have a query about it, and to use the call center. Younger women may also face additional barriers to accessing MR facility services due to age, childbearing, and partnership norms [36].

### Limitations

Our study has some limitations and implications for further research. We collected limited data on repeat callers, and these were not linked, so the characteristics of the 45% of calls who are repeat callers are overrepresented in the data. It would be useful to understand more about repeat callers: why they called more than once and the duration between calls, for example. Further information is also needed about non-MR callers, as the numbers of these have been increasing and now account for a quarter of calls. This could suggest a further need for accessible advice for other reproductive health issues such as family planning or a change in the way the call center is promoted to better address other reproductive health needs. Further research is needed to understand the impact of this intervention on provider provision practices and client outcomes such as complete MR, use of post-MR family planning, and access to appropriate treatment for complications.

### Conclusions

Evidence has demonstrated that availability of misoprostol can lead to a decrease in the rate and severity of abortion complications [4]. To achieve this, a comprehensive harm reduction approach is needed; the key components include procuring and distributing high-quality drugs, ensuring training and support mechanisms for providers, building effective referral mechanisms for complication management, and addressing the need for postabortion family planning. This study demonstrates that a call center can form an important part of such a harm reduction package by providing information to providers and MR users and advising on post-MR family planning.

## Abbreviations

DHS: Demographic and Health survey
ITS: interrupted time series
LMIC: low- and middle-income country
mHealth: mobile health
MR: menstrual regulation
MSB: Marie Stopes Bangladesh
MVA: manual vacuum aspiration
WHO: World Health Organization
