# Title Correction: Using a Call Center to Reduce Harm From Self-Administration of Reproductive Health Medicines in Bangladesh: Interrupted Time-Series

**DOI:** 10.2196/15902

**Published:** 2019-08-19

**Authors:** Katherine Keenan, Katharine Footman, Munnaf Sadekin, Kate Reiss, Reena Yasmin, Hannah Franklin, Kathryn Church

**Affiliations:** 1 University of St. Andrews School of Geography and Sustainable Development St. Andrews United Kingdom; 2 Marie Stopes International London United Kingdom; 3 Marie Stopes Bangladesh Dhaka Bangladesh; 4 London School of Hygiene and Tropical Medicine London United Kingdom

The title of this paper (JMIR Public Health Surveill 2019;5(3):e12233) has been changed from “Using a Call Center to Reduce Harm From Self-Administration of Abortion Medicines in Bangladesh: Interrupted Time-Series” to “Using a Call Center to Reduce Harm From Self-Administration of Reproductive Health Medicines in Bangladesh: Interrupted Time-Series”.

The title was changed by the editorial office to enhance clarity. The authors are concerned that the edited title suggested that the call center supports abortions, which is not the case. The call centre supports administration of menstrual regulation medications. The authors also wish to emphasize that Marie Stopes Bangladesh does not provide abortion medications. The organization provides menstrual regulation services

The corrections will appear in the online version of the paper on the JMIR website on August 19, 2019, together with the publication of this correction notice. Because this was made after submission to PubMed and Crossref, the corrected article also has been resubmitted to those repositories.

